# Case Report: Lateral Habenula Deep Brain Stimulation for Treatment-Resistant Depression

**DOI:** 10.3389/fpsyt.2020.616501

**Published:** 2021-01-13

**Authors:** Zhiyan Wang, Xiaodong Cai, Rirang Qiu, Chen Yao, Ye Tian, Chen Gong, Yingli Zhang, Bin Xu, Doudou Zhang, Yu Zang, Jiali Liu, Bo Peng, Luming Li

**Affiliations:** ^1^National Engineering Laboratory for Neuromodulation, Tsinghua University School of Aerospace Engineering, Tsinghua University, Beijing, China; ^2^Department of Neurosurgery, Shenzhen Second People's Hospital, the First Affiliated Hospital of Shenzhen University, Shenzhen, China; ^3^Department of Depressive Disorder, Shenzhen Mental Health Center, Shenzhen, China; ^4^Precision Medicine and Healthcare Research Center, Tsinghua-Berkeley Shenzhen Institute, Tsinghua University, Shenzhen, China; ^5^IDG/McGovern Institute for Brain Research at Tsinghua University, Beijing, China; ^6^Institute of Epilepsy, Beijing Institute for Brain Disorders, Beijing, China

**Keywords:** lateral habenula, patient, local field potentials, deep brain stimualtion, treatment resistant depression

## Abstract

Treatment-resistant depression (TRD) is a chronic and severe psychiatric illness associated with limited therapeutic options. Deep brain stimulation (DBS) is a promising therapy for TRD patients. However, its safety and efficacy are still unclear. Here we reported the safety and efficacy of lateral habenula (LHb) DBS for a TRD patient who had failed medical, psychological, electroconvulsive, and ketamine therapy. The DBS system is compatible with 3T magnetic resonance imaging along with local field potential (LFP) streaming. Two DBS electrodes were implanted at the bilateral LHb without any complication. The patient showed acute stimulation effects and achieved long-term improvements in his depression, anxiety, and sleep with left LHb 160 Hz frequency stimulation, accompanying the change of LFPs. These results provided clinical evidence toward the safety and efficacy and electrophysiological basis of LHb DBS for TRD.

## Introduction

Depression is a chronic and severe psychiatric illness, affecting up to 300 million individuals worldwide (1). Approximately 30% of patients with depression fail to respond to two or more standard antidepressants with adequate doses and duration, which indicates the presence of treatment-resistant depression (TRD) (2). Deep brain stimulation (DBS) is becoming a promising therapy for TRD. Clinical studies have assessed putative therapeutic effects of DBS in participants with TRD across several major brain targets, such as subgenual cingulate gyrus (SCG), ventral anterior limb of internal capsule (vALIC), superolateral medial forebrain bundle (slMFB), and lateral habenula (LHb) (3–6). However, the efficacy and safety of these techniques are still unclear (7–9).

The LHb is a phylogenetically old structure located in the dorsomedial portion of the thalamus, which is an important link between the forebrain and brainstem monoaminergic nuclei (10). Due to the long-standing monoaminergic hypothesis of depression, the LHb is identified as a potential target for DBS (11). Sartorius et al. (6) has first reported a major depressive patient who achieves remission after a 12-week DBS of LHb. However, it is not certain whether the treatment is effective or accidental. The role of LHb in the pathogenesis of depression is described in detail, namely, an increase in the number of burst-firing neurons until 2018 (12). This provides a preclinical theoretical basis for high frequency DBS to treat TRD patients.

Our male patient was 34 years old and was enrolled in the clinical trial for TRD with the DBS of LHb (ClilnicalTrials.gov identifier: NCT03667872) in January, 2019. He suffered from depression beginning at the age of 13 without any hypomanic or manic episodes. In the past 21 years, he had experienced five major depressive episodes, accompanied by severe depression, loss of interest, and abnormal sleep disorders. Multiple pharmacotherapeutic trials involving antidepressants and augmentation with selective serotonin reuptake inhibitors, serotonin and norepinephrine reuptake inhibitors, and other antipsychotic medications could not control his depression, which resulted in substantial functional impairment (e.g., long-term disability from work) in recent years.

Two years ago, he tried six intravenous ketamine to treat his disease. The depressive symptoms had eased but not reached to the normal level, and he relapsed 3 months later. Then he received the electroconvulsive therapy (ECT). Although remission was achieved after ten sessions, the effects cannot be maintained and the patient cannot bear its side effects. The patient gave informed consent for participation in the clinical trial, which was approved by Shenzhen Second People's Hospital and Shenzhen Kangning Hospital Ethics Board. Two independent psychiatrists evaluated his psychotic symptoms with 21-item Hamilton Depression Rating Scale (HDRS_21_), which was 24, and still was 23 a month later. During this month, the doctor performed a detailed physical examination, a series of mental scale assessments and MRI examination on him in accordance with the inclusion and exclusion criteria, and ruled out other psychiatric diagnoses in the diagnostic and statistical manual of mental disorders-fifth edition (DSM-5).

## Materials and Methods

### Surgery

Both preoperative and post-operative high-resolution magnetic resonance imaging (MRI) images were scanned with 64 channel RF head coil (3.0T; SIEMENS MAGNETOM Prisma_fit, Erlangen, Germany). Two sets of 0.7 mm isotropic T1_mprage and SWI images were obtained, respectively, at 1 month and 1 day before the surgery. A group of 128-directions 2 mm isotropic diffusion images with opposite phase encoding direction was acquired for connectomic analysis. The high-resolution anatomic MR images delineated the habenula clearly in 3-D space.

A CT scan with bone markers anchored into the skull of the patient was scanned and merged on the morning of the surgery day. Under general anesthesia, two burr holes were drilled continuously on both sides. Immediately after the registration of ROSA robotic arm (Medtech, Montpellier, France) for the second time, the dura mater and pial of one side was penetrated by a cannula and a quadripolar DBS electrode with 0.5 mm spacing between each contact (L301c, PINS, Beijing, China) was implanted into the target without micro-electrode recording. A successful introperating CT showed that DBS electrodes were implanted into targets as planned on both sides with few pneumatosis in the prefrontal lobe. Two extensions (E202c, PINS, Beijing, China) and a pulse generator (G102RS, PINS, Beijing, China) were implanted subsequently. No intracerebral hemorrhage was found for 24 h after operation. Besides, 3T-MRI scans were performed at 1 and 2 m after surgery to observe whether the electrodes were displaced.

### Clinical Evaluation and Follow-Up

**Figure 1:**
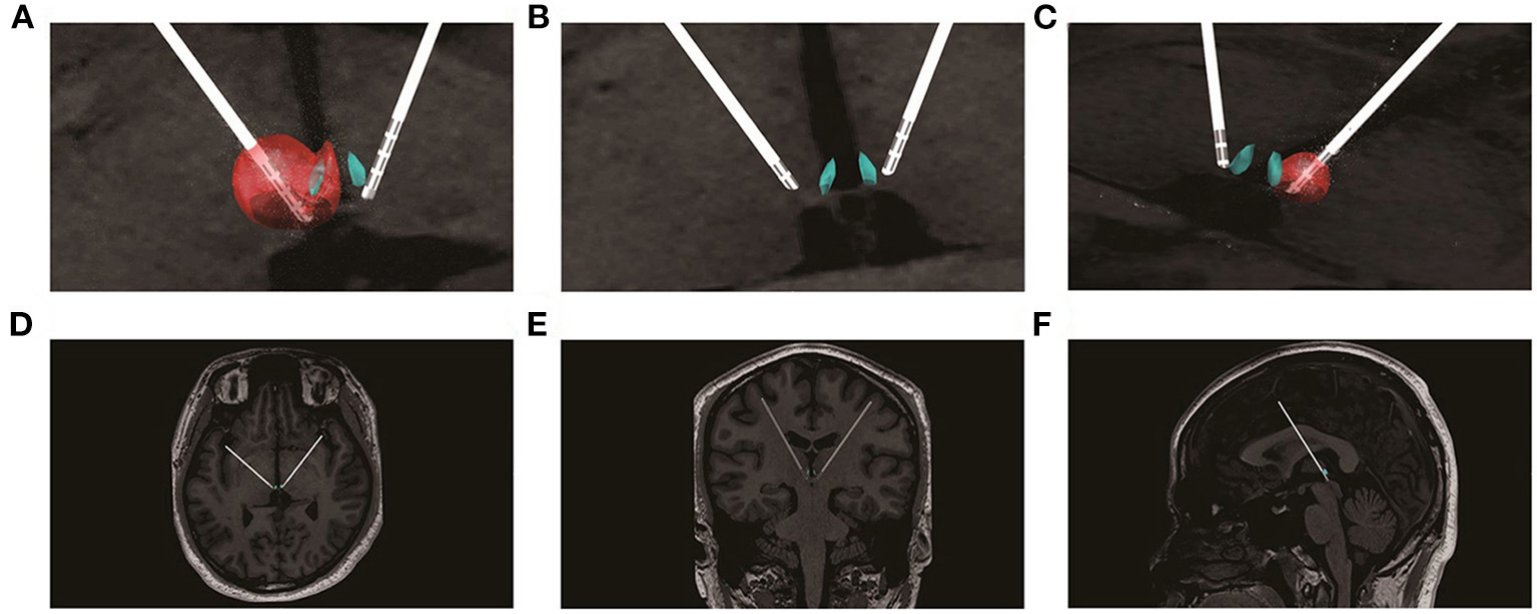
Lead placement, electric fields, and estimated volumes of tissue activated for the stimulation settings post-implantation. **(A)** the estimated volumes of tissue affected for the left side stimulation settings (monopolar stimulation, L3-, 4.5 v, 160 Hz, 90 μs); **(B)** enlarged view of the electrode positions; **(C)** the estimated volumes of tissue affected for the right side stimulation settings (monopolar stimulation, R3-, 2.5 v, 160 Hz, 90 μs); **(D)** the electrodes in the transverse section; **(E)** the electrodes in the coronal section; **(F)** the electrodes in the median sagittal section.

The program testing was carried out 3 weeks post-surgery. Firstly, from post-op MRI, we calculated the stimulation voltages that could cover the left and right LHb, respectively, by using SimBio/FieldTrip model (13) through LeadDBS version 2.2.0. As shown in Figure 1, the best estimated stimulation voltages were 4.5 and 2.5 v of the left and right LHb, respectively. The patient was feeling well without discomfort using the asymmetric calculated parameters. Therefore, the tested parameters (monopolar stimulation, frequency = 160 Hz, pulse width = 90 μs, voltage left = 4.5 v, voltage right = 2.5 v) were used for acute test stimulation.

The acute stimulation protocol consisted of 12 trials (one at each of the 8 available contacts; 4 left, 4 right plus 4 sham trials) of 3 min stim-on followed by 3 min stim-off. the stimulation voltages of left and right contacts remained the same during the process. The order of active or “sham” trials were randomized and the clinician assessor were blinded to the condition. The patient was instructed to describe himself during each trial for any changes in sensation, feelings, mood and thoughts. Self-reports were recorded at fixed time points within each trial (1 min after initiation of stimulation and again 1-min following discontinuation of stimulation). At conclusion of the protocol, responses for each of the 12 trials were reviewed (Table 1). In the follow-ups, we adjusted the bilateral LHb stimulation voltages within 0–10 V. The subsequent stimulation voltages were determined by both the efficacy of the patient's previous parameters and the responses of this adjustment parameters.

**Table 1:** Narrative descriptions recorded our participant.

**Random order**	**Contact**	**Response (Self-report)**	
		**DBS OFF**	**DBS ON**
1	Left 3	“It's difficult to think” “I feel numb”	“Feel better than the previous one” “10% improvement than yesterday”
2	Left 4	“I feel a little boring, numb, and no freshness”	“Feel a little better” “Speak faster”
3	Left 2	“My head reacts slowly”	“Not good than the previous trial” “My mood feels a little worse than yesterday”
4	Sham 2	“I feel not good” “My head reacts slower” The worst one	“No change”
5	Right 1	“I feel not good and a little depressed”	“I feel better than the previous one and worse than the first and second trial”
6	Sham 3	“Speak slowly”	“No change”
7	Left 1	“I feel not good and a little depressed”	“I feel better than the previous one, but still react a little slow, and inattention”
8	Right 2	“My head reacts slowly” “I feel no motivation and depressed”	“No significant change”
9	Right 4	“I feel a little depressed” “My mood is worse than yesterday”	“No change”
10	Right 3	“Feel worse than the previous one”	“No change”
11	Sham 1	“I feel a little depressed”	“No significant change”
12	Sham 4	“I feel a little depressed”	“No significant change”

Long-term clinical efficacy was evaluated by the psychiatrists who were blinded to the current stimulus parameters and/or changes. Evaluation included the HDRS_21_ versions, the Montgomery Asberg Depression Scale (MADRS), the Quick Inventory of Depressive Symptomatology-Self-Report (QIDS-SR), the Hamilton Anxiety Rating Scale (HARS), the Pittsburgh sleep quality index (PSQI), the Hypomania Symptom Checklist-32 (HCL-32), the frontal assessment battery (FAB), and the Short Form Health Survey (SF-36). As a baseline assessment, the ratings were performed at enrollment and 1 week prior to surgery. The DBS stimulation was done 3-weeks after surgery. The same ratings were performed at 1, 4, and 12 weeks after the stimulation. Medications were unchanged throughout the follow-up period.

### LFP Acquisition and Data Analysis

The LFP electrodes were the DBS leads. These quadri-contacts cylindrical leads had 1.5 mm long contacts with 0.5 mm distance in between. All the LFP signals were sensed differentially through adjacency contacts by implantable pulse generator (IPG) with 1,000 Hz sampling rate. The detailed methods of the LFPs data collection and processing were described in our previous studies (14). The 10-min data was collected when the stimulation was off at each time. It was processed by a 0.5–50 Hz band-pass filter and segmented to 1 s length. Power spectrum density (PSD) of each 1 s epoch was generated by Welch's method with a Hamming window of 0.2 s and an overlap of 75%. PSD in the five typical bands (delta band, 1–4 Hz; theta band, 4–8 Hz; alpha band, 8–15 Hz; beta band, 15–30 Hz; part of gamma band, 30–50 Hz) was calculated using SciPy library of Python.

## Results

### Acute Clinical Outcomes

The patient reported that his reaction and negative mood had a 10% improvement with the stimulation at the third contact of the left side (monopolar stimulation, 4.5 v, 160 Hz, 90 μs), and it disappeared when the stimulation turned off. This effect was not significant during testing of other contacts. As a result, the initial programming was dominated with the left side stimulation (L3−, 4.5 v, R3−, 2.5 V; C+, 160 Hz, 90 μs).

### Long-Term Clinical Outcomes

**Figure 2:**
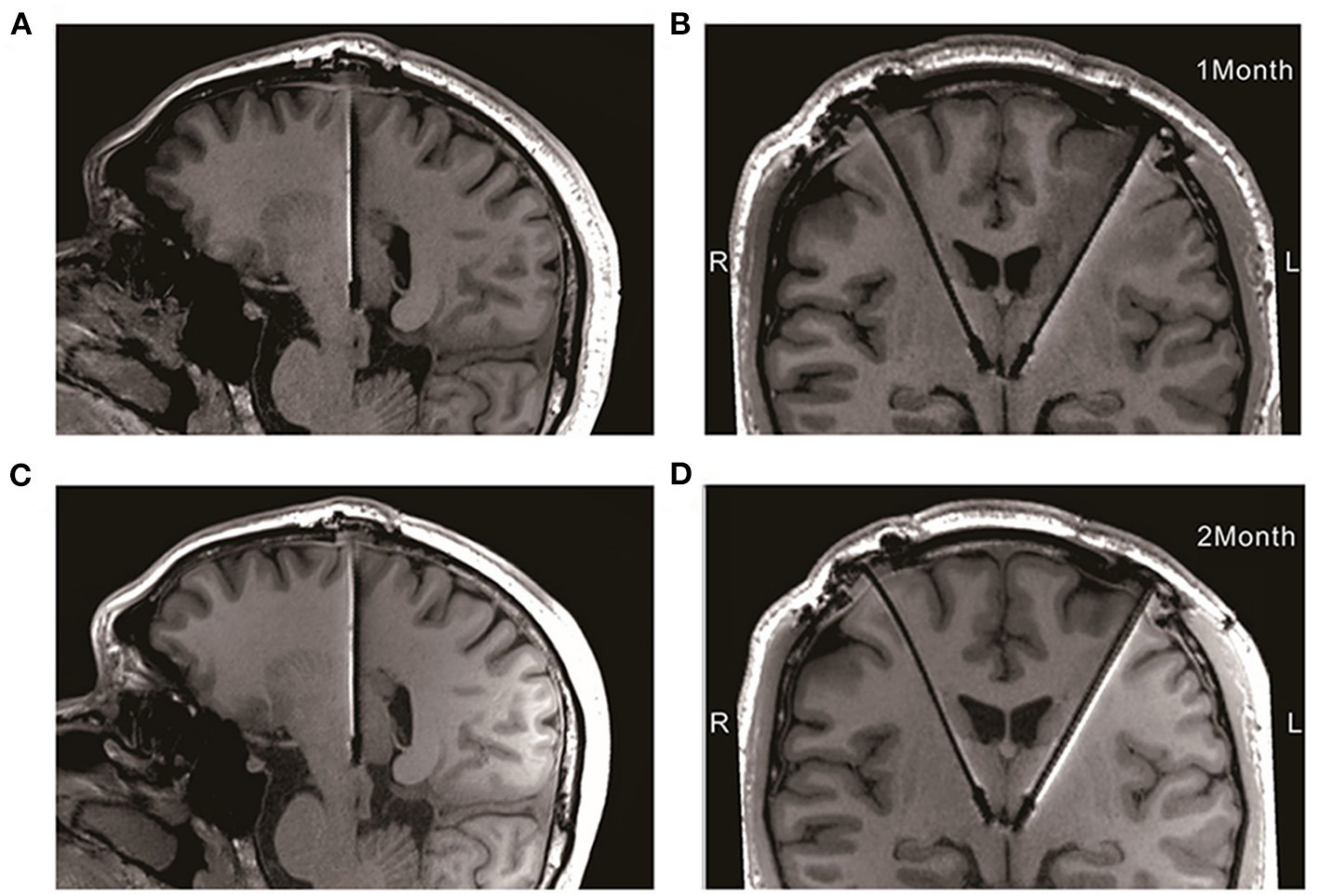
Electrodes placement at 1 and 2-month after implantation with 3T MRI. **(A,B)** The electrodes in the median sagittal and coronal section at 1-month after implantation, respectively; **(C,D)** The electrodes in the median sagittal and coronal section at 2-month after implantation, respectively.

During the 12-weeks follow-ups, his negative mood, anxiety, sleep quality, and quality of life showed noteworthy progressive improvements with DBS of the LHb (Table 2). He also reported better concentration, less impatience and increased libido than previously. High frequency of the LHb did not impact the patient's cognitive functions based on Frontal Assessment Battery test. Moreover, stimulation did not induce adverse side effects related to the surgery or chronic stimulation, except for dizziness during the stimulation onset, which was reversible. The parameters underwent fine tuning: the left side stimulation increased to 5.0 v (1 week, 4.5 v; 4 weeks, 5 v; 12 weeks,5 v) and the right side stepwise switched off (1 week, 2.5 v; 4 weeks, 1.5 v; 12 weeks, 0 v), the frequency and pulse width remained constant. Moreover, no electrodes displacement occurred at 1 and 2-month after implantation with 3T MRI (Figure 2).

**Table 2:** Clinical outcome measures.

**DBS status**	**Pre-op**	**HFS**	**HFS**	**HFS**
**Rating scale**	**Baseline**	**1 wk**	**4 wk**	**12 wk**
HDRS_21_	23	12	9	10
MADRS	29	13	6	10
QIDS-SR	19	6	4	7
HARS	20	6	8	7
Somatic	8	2	2	2
Mental	12	4	6	5
PSQI	14	10	8	1
HCL-32	7	1	1	1
FAB	17	18	18	18
SF-36				
Physical functioning	95	95	95	95
Body pain	62	94	84	100
Social functioning	50	50	62.5	75
Mental health	32	36	32	44
Reported health transition	75	75	75	75

*Pre-op, pre-operative; HFS, high frequency stimulation*.

Approximately 18 weeks after stimulation, he had to quit the further observation for his legal responsibility, which was not associated with DBS stimulation.

### LFP Outcomes

**Figure 3:**
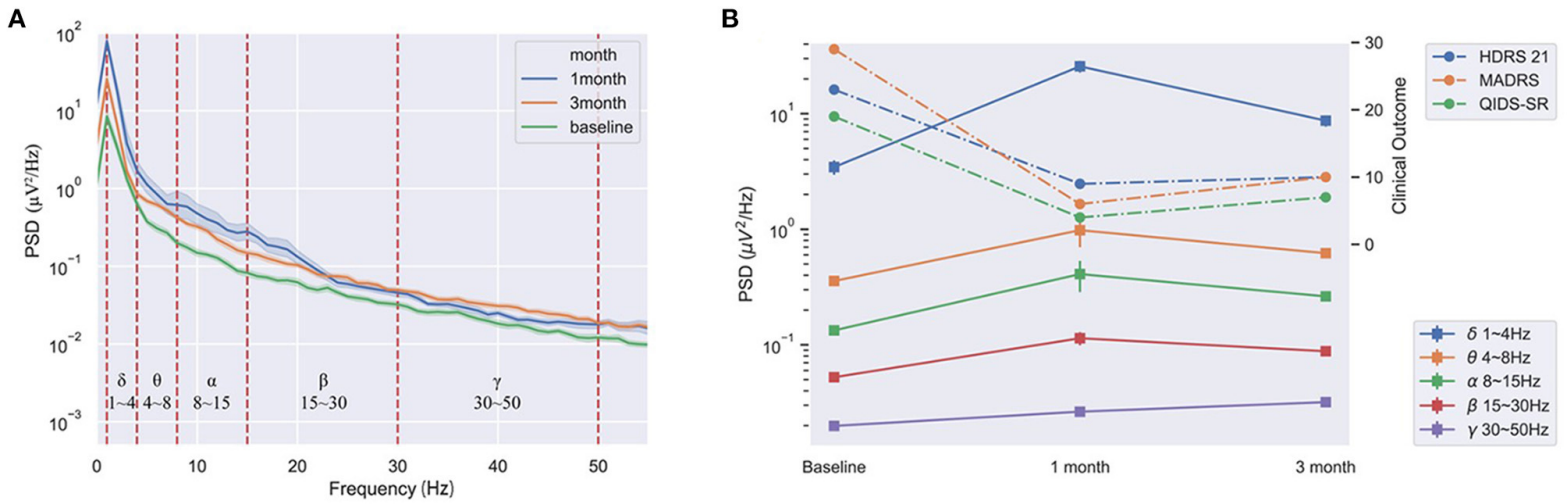
The consistent trends of the PSD at DBS off of the LHb and the clinical improvements of the patient at pre-operation, 1 and 3-month after stimulation. **(A)** The PSD at DBS off in all bands at pre-stimulation, 1 and 3-month after stimulation. **(B)** The average PSD had positive and consistent trends between the delta, theta, alpha, and beta bands and the clinical improvements at pre-operation, 1 and 3-month.

The PSD at the DBS off of LFPs had positive and consistent trends between lower frequency bands and the clinical improvements, including delta, theta, alpha, and beta bands. The better the clinical improvements of the patient, the higher PSD in these frequency bands (Figure 3).

## Discussion

Our study reported that a TRD patient obtained acute stimulation effects and long-term clinical improvements with LHb DBS stimulation using an advanced DBS system. Moreover, With the clinical improvements, the PSD of LFPs gradually increased in lower frequency bands.

Our case first highlighted the acute antidepressive effects of high frequency DBS of LHb for TRD. Although a minor response of the “salient” contact stimulation, it indicated a rapid response in mood-regulatory circuits with DBS of LHb. Furthermore, the same DBS stimulation resulted in sustained improvement of depressive symptoms in our patient. The acute behaviors of the patient in LHb DBS may provide a potential biomarker for predicting the clinical effects of chronic stimulation.

Our case also first reported the electrophysiological change of high frequency DBS of LHb for TRD. The LFPs results implied the LHb cellular activities increased when the stimulation was off, and this change was related to the improvement of clinical depression symptoms. Animals studies found that attenuating LHb hyperactivity using DBS ameliorated learned helplessness and maternal separation and animals' depressive-like symptoms (15, 16). What is more, Clemm von Hohenberg et al. (17) found a causal link between LHb downregulation and reduction in DMN connectivity. Our LFP results appeared to conflict with previous findings in animal models that LHb hyperactivation was associated with depression (12, 15, 16, 18). It may be that the psychopathology of depressive patients is highly complex and the animal models cannot be fully modeled (19). However, no studies have reported the physiological characteristics of patients with depression. Our clinical study is ongoing and more data collection could reveal the electrophysiological changes of LHb DBS in TRD patients.

LHb was known to exert a powerful control over the midbrain dopaminergic ventral tegmental area and substantia nigra pars compacta and serotonergic dorsal and median raphe, and GABAergic rostromedial tegmental nucleus (10, 20, 21). Animal studies found that LHb DBS significantly improved depressive-like symptoms and increased the concentration of monoamines including dopamine and serotonin in blood serum and brain tissue (22). This evidence suggests that chronic DBS stimulation may induce neural plasticity changes in habenular-associated neural circuits, which should be studied further.

The volume of the entire Hb is only 27 mm^3^, and it is located below the third ventricle (23). In our study, the DBS electrodes were implanted as precisely as possible, and no electrode displacement occurred after implantation, which was identified by the 3T MRI compatible electrodes after surgery. We used left (4.5 V) and right (2.5 V) LHb stimulations in our initial trials because of the asymmetry of the closet contacts to bilateral LHb. Recently, He et al. (24) reported the Hb could be visualized using susceptibility weighted imaging and quantitative susceptibility mapping, which could help electrode implantation to be more precise.

Furthermore, the left stimulation voltage was exclusively maintained at 5 V in our subsequent test, suggesting that the left-side stimulation is specifically effective in our patient. The result was contradictory to the study that ketamine induced a reduction of metabolism in the right habenula in TRD patients (25). However, other studies found that the habenula volume had higher volume on the left side in humans (26) and no main effect of hemisphere in major depression patients (27). These results suggested that the asymmetric role of LHb needs further confirmation. Therefore, it cannot be excluded that the same stimulation voltage applied to both sides of LHb would be more effective for TRD. Another limitation was that there was a lack of long-term efficacy and the optimal stimulation parameters observation due to the patient's cessation of further follow-up.

In conclusion, we are the first to report that an acute high frequency stimulation effect later engendered significant clinical improvement as well as its electrophysiology change in a TRD patient with DBS of LHb. Although there are some limitations in our study, the results prove that DBS of LHb is a safe and effective treatment for TRD.

## Data Availability Statement

The raw data supporting the conclusions of this article will be made available by the authors, without undue reservation.

## Ethics Statement

The studies involving human participants were reviewed and approved by Ethics Committee of Drug Clinical Trials of Shenzhen Second People's Hospital. The patients/participants provided their written informed consent to participate in this study. Written informed consent was obtained from the individual(s) for the publication of any potentially identifiable images or data included in this article.

## Author Contributions

ZW has designed the clinical trial, analyzed the data, and wrote the manuscript. XC has performed the DBS implantation and collected the data related to surgery. LL has provided the main financial support and guided the conduction of the clinical trial. YZh has recruited the patient, collected the data related to disease, and guided conduction of the clinical trial. CY, BX, DZ, and JL has collected the data related to surgery. RQ, YZa, and BP has collected the data related to disease. All authors contributed to the article and approved the submitted version.

## Conflict of Interest

The authors declare that the research was conducted in the absence of any commercial or financial relationships that could be construed as a potential conflict of interest.
